# Supplementary material for: Facilitating bioinformatics reproducibility with QIIME 2 Provenance Replay
Source: PLoS Comput Biol. 2023 Nov 27;19(11):e1011676. doi: 10.1371/journal.pcbi.1011676 (PMC10703398; doi:10.1371/journal.pcbi.1011676)
Supplement: Attachment — Submitted filename: plos-comp-bio-response-to-reviewers.pdf [file pcbi.1011676.s002.pdf]

To the editor,
We have considered all of the reviewers' and editor's feedback and integrated changes to address this feedback in the current revision. We feel that this has substantially improved our manuscript.

Below is a point-by-point response to all of the feedback we received. The feedback received from the editor and reviewers is quoted in italicized text, and our responses follow inline.

In this revision we have added an author, Elizabeth Gehret, to the manuscript. Elizabeth developed our new version of Figure 2. I have included a PDF with the submission indicating approvals to add Elizabeth to the author list from all co-authors, as requested by the editors.

Thank you for the opportunity to revise and resubmit our manuscript to PLoS Computational Biology.

Best,
J Gregory Caporaso
31 October 2023

Editor feedback

I agree it will be important to reduce the reliance on the master's thesis by going back to the original citations and this would also be fair to the authors of these studies.

We have removed the reliance of this paper on the original master's thesis by migrating essential content to this manuscript. We have also ensured that the master's thesis is easily accessible (now on FigShare, with a DOI that is cited in this manuscript) in response to the reviewers' feedback on the difficulty of accessing it.

Regarding the tension between developing the concepts about reproducibility and explaining the actual work sufficiently, I agree that the latter is essential but if the authors can manage to better explain the conceptual work in a concise way, I would be happy to have that included. However it must become clearer than it is at the moment. I felt the explanation was not clear enough for someone not familiar with the ideas already and for those who already are, it is less of a problem but not doing a great job as to what are new ideas and why they are important. If you decide to develop this into a separate publication that is fine. If you want to keep it, make it clearer while keeping it concise.

We have reworked our discussion of reproducibility concepts, and used it to better contextualize the software presented here.

Reviewer #1 feedback

The presented software article motivates and presents a python application capable of extracting metadata from a QIIME2 analysis. This metadata serves as documentation of the analyses performed within QIIME2, and can be used to re-run the analyses. The advantage of programmatically extracting this documentation, or data provenance information, is that it requires very little time and effort from the researcher and guarantees retrieval of accurate and complete information, which can be tedious to achieve manually.

The application is a useful and much needed addition to the QIIME2 software suite, making it easier and faster for researchers to provide provenance information in scientific publications and related works. The idea is not

particular novel, and references to exiting work need to be added, but means to increase the chances that scientists provide good provenance data are very important.

The presentation of the application, its output, and the placement in the field should be improved before publication as outlined in the major comments below.

Major comments:
1- The last sentence of the abstract says '… and discuss considerations for bioinformatics developers who wish to implement similar functionality in their software.' I cannot find this back in the Results or Future Directions sections. I think it would be useful to list the key elements that should be reported for microbiome research, and any suggestions you might have for developers in the field.

We thank the reviewer for pointing out this omission. We have added this discussion to the *Availability and Future Directions* section.

2- The manuscript heavily relies on the reference Keefe 2022, a master thesis which is not easily accessible, only upon request. I feel this is a major limitation and all information necessary to fully understand the paper, its motivation and the application should be given in the manuscript or supplement.

We apologize for the trouble accessing the thesis. We have addressed this feedback in two ways. First, we have uploaded the thesis to FigShare, and it is now accessible under DOI: https://doi.org/10.6084/m9.figshare.24217224.v1. Second, we have reduced our dependence on the thesis, migrating ideas that we were referencing to new content in this manuscript. We now only reference the thesis one time in the manuscript, referencing where more information can be found on our requirements engineering process.

3- No proper introduction of the field of workflow documentation is given, and references to similar approaches are missing. This needs to be added. For example, Snakemake generates an analysis graph (DAG) and provides documentation during runtime, the config file allows 'replay' at any time. Other post-analysis documentation tools exist but are not discussed and cited. See how Love et al, 2020, provided an overview over existing applications for provenance tracking of RNA-seq data for how it should look like.

We have expanded our discussion of workflow documentation to cover tools including Jupyter Notebook, Snakemake, and RMarkdown, including highlighting some pitfalls of these approaches. We additionally have added discussion of related tools and efforts in a new paragraph in the *Introduction* section, including a reference to the Love et al 2020 paper's review of related tools.

4- I like the reference to the reproducibility classes of the Turing Way categories/Gundersen. The figure legend (figure 1) is a bit hard to digest though. The concept is clear and easy to understand, so make the figure legend easy to comprehend as well, cite the references in the legend as well, and keep the details for the main text.

We have rephrased the Figure 1 caption, and had all authors review this content for readability. We thank the reviewer for pointing out the confusion.

5- The authors do not place their application in the context of Figure 1. Please do so and discuss the challenges associated with the more general reproducibility classes.

We have addressed this with new text in both the *Introduction* and the *Availability and Future Directions* sections.

6- Figure 2 is extremely difficult to interpret with the limited information given. Even if the reader is very familiar with the steps of the analysis, it is impossible to match input and output data and actions/methods to the DAG. The DAG needs to be sensibly annotated to be useful. Same holds for the Action details which are almost not human-readable in the presented form. Make sure the action details are easily matched to the DAG and the input/output files of the user, to make the DAG a useful feature of Provenance Replay.

In response to this feedback we have completely redesigned Figure 2. We believe this should address the concerns raised by the reviewer.

Minor comments:
1- Avoid spoken expressions like "aren't" in the text (abstract).

We have addressed this issue in the abstract, and reviewed the full text to formalize the writing where necessary.

References:
[Love et al., 2020] Love MI, Soneson C, Hickey PF, Johnson LK, Pierce NT, et al. (2020) Tximeta: Reference sequence checksums for provenance identification in RNA-seq. PLOS Computational Biology 16(2): e1007664. [*https://doi.org/10.1371/journal.pcbi.1007664*](https://doi.org/10.1371/journal.pcbi.1007664)

Reviewer #2 feedback

Reviewer #2: Keefe et al have developed a useful software component that will, as advertised, serve as an aid to reproducibility in microbiome data analysis. The problem that the authors identify is real: even sophisticated bioinformatics scientists have trouble recording and documenting complex analytical workflows. The solution presented by the authors is to let the computer record the steps and software dependencies from the analysis, a task which seems to be so much more appropriate for computers than humans. While this collusion only covers one field of research on one bioinformatics platform, it does point the way forward for other areas of research and software.

Major comments
1. There is a tension within the article between developing broad ideas about reproducibility vs introducing the software. As the primary purpose of this article is to introduce new software, the authors should not develop broad ideas that are not absolutely necessary to motivate and describe the software presented. As just one example, is it necessary to merge reproducibility categories from two organizations into a unified hierarchy? Can't the software's utility be justified separately in the context of each framework, without merging them? Personally, I think the broad ideas are interesting and would make for an impactful, but separate, manuscript. The authors do not have the space to properly develop broad ideas about reproducibility here, and any attempts to do so will distract from the main focus of the paper.

In response to this feedback, and the editor's note that we could choose to retain this information if this discussion was better motivated and clarified, we have reworked this text and the corresponding figure legend to simplify this discussion and use it to contextualize the software that we are presenting.

2. Much of the background for this manuscript seems to come from a Master's thesis written by the lead author. The thesis is primarily used as a source for the goals that the software seeks to meet. I tried but was unable to obtain a copy of the thesis from the library at Northern Arizona University. I understand referencing a thesis, but am uncomfortable with the degree to which the basis of the article relies on a thesis from the lead author. As I've not read the thesis, I'm not sure about the degree to which the goals are crafted by the author himself vs. aggregated from other sources. If the goals outlined in O'Keefe 2022 are collected from other sources, please cite them directly. Throughout the article, the reliance on O'Keefe 2022 should be minimized in favor of briefly describing the rationale and citing original sources.

We apologize for the trouble accessing the thesis. We have addressed this feedback in two ways. First, we have uploaded the thesis to FigShare, and it is now accessible under DOI: https://doi.org/10.6084/m9.figshare.24217224.v1. Second, we have reduced our dependence on the thesis, migrating content that we were referencing to this manuscript. We now only reference the thesis one time in the manuscript.

3. In the Design and Implementation section, the authors say, "initial software design was based on literature review, existing API targets, and discussion with QIIME 2 developers, as well as an initial requirements engineering process and formal focus groups with prospective users." Even if you don't have space for all the details, we at least need to hear about the requirements engineering process, and we need to see some data from the focus groups. Because you have gone above and beyond the design practice in many labs, the readers need to see what a robust design process looks like. And if you did it right, these data will provide strong support for your design.

We thank the reviewer for this suggestion. We have added a new paragraph providing additional detail on this process, and this is the one location where we retained a reference to the original thesis, which contains a lot of additional detail. We have ensured that the thesis will be easily accessible to readers interested in following the reference.

4. In Availability and Future Directions, the authors say, "this approach can provide a model for other scientific computing platforms," but then don't address HOW other computing platforms might use ideas from QIIME 2 Provenance Replay. This is the one glaring question that needs to be addressed by the authors. It seems to me that the design was so successful in QIIME 2 because the framework was built from the ground up to keep track of software used in each step. Is the plan to decouple QIIME 2 from microbiome data analysis and extend into other areas? To have separate QIIME 2-like frameworks for each field of research? To develop a general tool that tracks software dependencies on the command line? How does Galaxy fit into this picture? Do Jupyter Notebooks and RMarkdown have anything to learn from this? If you have to trim other parts of the article to make space for exploring this question, please do. This is where you can emphasize the payoff from your software and examine some broad ideas while staying relevant to the topic of the article.

We thank the reviewer for pointing out this omission. We have added discussion of how other platforms could use these ideas to produce similar functionality to the *Availability and Future Directions* section. We have additionally expanded our discussion of QIIME 2's expansion into other research domains, motivated in large part by research software engineers' interest in QIIME 2's data provenance and Provenance Replay. We also added new discussion of Jupyter Notebooks and RMarkdown in the *Introduction* section, and refer back to that discussion in the new text added to the *Availability and Future Directions* section in response to this same comment.

Minor comments

1. The reproducibility hierarchy introduced by the authors, merging work from Gunderson and Kjensmo with ideas from the Turing Way, is presented in an overly technical way. Instead of "we contextualize key factors in generalizability within the broader goals of reproducible research," you could just as easily say "we developed an expanded hierarchy that includes ideas from both groups." The figure legend is impossible to understand without a full understanding of the text, which is itself difficult. This prevents the figure from serving as a visual introduction to your ideas about reproducibility. This may be a moot point if you follow the suggestion from major comment 1.

We have addressed this by re-working the text introducing these ideas, and by rewriting and simplifying the figure caption.

2. You probably mean to cite the Turing Way as "The Turing Way Community. (2021, November 10). The Turing Way: A handbook for reproducible, ethical and collaborative research. Zenodo. http://doi.org/10.5281/zenodo.3233853"

We thank the reviewer for catching this error. We have fixed this citation.
